# Supplementary material for: Manganese and iron deficiency in Southern Ocean Phaeocystis antarctica populations revealed through taxon-specific protein indicators
Source: Nat Commun. 2019 Aug 8;10:3582. doi: 10.1038/s41467-019-11426-z (PMC6687791; doi:10.1038/s41467-019-11426-z)
Supplement: Supplementary file 1 — Supplementary Information [file 41467_2019_11426_MOESM1_ESM.pdf]

Supplementary Information for “Manganese and iron deficiency in Southern Ocean *Phaeocystis antarctica* populations revealed through taxon-specific protein indicators”

Wu, McCain et al. Nature Communications 2019

Supplementary Discussion

Two main themes regarding protein expression patterns warrant further discussion and are highlighted below. These include protein expression changes relating to reactive oxygen and those relating to carbon allocation and acquisition.

Changing light and metal conditions can have dramatic consequences for reactive oxygen species (ROS) production within photosynthetic cells. We found a variety of expression patterns across metal treatments for different ROS metabolizing and redox-related proteins. We found no evidence of differential expression across treatments of ROS enzymes related to the antioxidant glutathione (i.e. glutathione peroxidase and two glutathione S-transferases showed no differential expression across metal conditions) except nucleoredoxin. Further, nickel superoxide dismutase (NiSOD) was similarly expressed across treatments. A potential ROS-metabolizing protein with differential expression was peroxiredoxin, which was upregulated only under low Mn and low Fe. Interestingly, peroxiredoxin can play a dual role of ROS metabolism and cell redox signaling[1,2]. Overall, we observed several additional signs of oxidative stress in the low Mn low Fe condition (discussed in main text), while some ROS metabolizing proteins also may play a regulatory role.

Thioredoxins, a group of proteins connected to electron flux in electron transport chains, showed differential expression across treatments. NADPH-dependent thioredoxin reductase was upregulated only in -Fe+Mn. Previous work has shown that thioredoxins can regulate carbon metabolism in diatoms[3], so the sustained production of NADPH in high Mn and low Fe was potentially directed to this regulatory protein. In contrast, a disulfide-isomerase domain-containing thioredoxin, was downregulated in the same condition. Two other thioredoxin-like

proteins showed no differential expression across metal treatments (Figure 2 and Supplementary Data 1).

We suggest that the phenotypic and proteomic differences observed across Mn and Fe treatments may be controlled in part via thioredoxins, given the variable expression profiles of this protein group. Thioredoxins are widely recognized as regulators of photosynthesis, carbon metabolism, and a central hub of redox-based protein regulation in plants[2], and likely play a similar role in diatoms[3]. Recently, thioredoxin-mediated control of chlorophyll biosynthesis has been documented in higher plants[4], and is a potential mechanism connecting electron flux, Fe, and Mn demand within phytoplankton. We suggest that the role of thioredoxin, peroxiredoxin and nucleoredoxin in regulation of key *Phaeocystis* metabolisms would be a fruitful line of future research.

Phosphoribulokinase (PRK), a key Calvin cycle enzyme[5], showed the highest expression in our intermediate irradiance treatment (Supplementary Figure 6), which is consistent with previous reports of maximum specific carbon fixation rate of *P. antarctica* at this light level[6]. Glycolytic enzymes (endolase and pyruvate kinase) increased the expression under increased light, suggesting higher rates of glycolysis. Phosphoglycerate kinase (PGK), involved in both glycolysis and Calvin cycle, showed higher expression under low light. PGK was down-regulated under low Fe and low Mn, but not under low Fe alone, potentially consistent with reduced carbon metabolism under the combined low metal condition. Low Mn resulted in lower PGK expression, but this was not significant. These data suggest that Mn deficiency and irradiance both play a role in PGK expression for either glycolysis, the Calvin cycle, or both (Supplementary Figure 6).

Trace metal deficiency resulted up-regulation of aldolase (contig_109012_45_1228_+, catalysing F 1,6-P $\leftrightarrow$ G3P+ DHAP) in low Fe and low Fe and low Mn, similar to previous

observations under low Fe.[7] Metal deficiency down-regulated triose phosphate isomerase (TPI) contig_166753_157_979_-, catalysing reversible interconversion of the dihydroxyacetone phosphate and glyceraldehyde 3-phosphate (G3P). Together, these expression patterns suggest declined glycolysis, or increased gluconeogenesis, perhaps to compensate for the lower glucose generation and energy from photosynthesis due to the trace metal deficiency. Further, we also observed down regulated pyruvate dehydrogenase and pyruvate carboxylase which transform pyruvate, produced in glycolysis, into acetyl-CoA and oxaloacetate (OAA), respectively. This suggests that *P. antarctica* cells in the -Fe and -Mn condition were experiencing declined metabolism.

Parallel to glycolysis is the pentose phosphate shunt. Enzymes in the pentose phosphate pathway detected in our proteome data could not be used for statistical analysis because they were not detected in all channels. However, 6-phosphogluconate dehydrogenase (catalysing 6-P-gluconate to R5P, producing NADPH) had higher abundance in low Fe, low Mn and low Fe and low Mn treatments, as shown in raw data (Supplementary Data 2). This was one potential pathway induced by Fe and Mn deficiency: G-6P could enter to produce R5P as substrates for supporting biosynthesis in the cell (Supplementary Figure 6).

Notably, an uncharacterized alpha/beta hydrolase was significantly up-regulated under low Fe and Low Mn (Figure 2B and Supplementary Figure 6), suggesting that cells may be hydrolyzing more complex compounds, rather than glucose, for obtaining energy and carbon. Further characterization of this unknown alpha/beta hydrolase may provide insights on the alternative energy and carbon acquisition mechanism of *P. antarctica* cells when experiencing low Fe and Mn conditions.

Supplementary Table 1

Mass spectrometry dataset descriptions and peptide spectrum matches (PSM)					
MS Datasets	Protein groups/Protein	Peptide group	PSMs	MS/MS spectrum	Quan Spectra
Run 1	1778/2182	3651	5902	122640	36759
Run 2	1574/1950	3093	4575	116320	31065

Supplementary Table 2 SRM parameters for peptides reported

Peptide name	Peptide (charge state)	Precursor (m/z)	Product (m/z)	Fragment ion	Collision Energy (V)	SRM rank (1: greatest signal)	RT (min)	LOD	LOQ
Phaeo Rubisco, small subunit 1	AKPNFYVK(+3)	322.85	409.24	y3	13	1	17.4	1.1	5.1
			556.31	y4	13	2			
			670.36	y5	11.6	3			
			767.41	y6	15.3	4			
	AKPNFYV(*13C15N*)K (+3)	324.86	415.26	y3	13	1			
			562.33	y4	13	2			
			676.37	y5	11.6	3			
			773.42	y6	15.3	4			
Phaeo Rubisco, small subunit 2	QIQYALNK(+2)	489.27	445.28	y4	16.4	2	12.5	0.1	0.7
			608.34	y5	15.8	1			
			736.40	y6	15.2	3			
			849.48	y7	17.7	4			
	QIQYAL(*13C15N*)NK (+2)	492.78	452.29	y4	16.4	2			
			615.36	y5	15.8	1			
			743.42	y6	15.2	3			
			856.50	y7	17.7	4			
Phaeo Plastocyanin 2	GGPHNVVFVEDAIPK(+3)	526.95	543.31	y5	15.1	4	28.7	0.1	1.0
			672.36	y6	13	2			
			771.42	y7	13.6	3			
			918.49	y8	14.7	1			
			1017.56	y9	16.9	5			
	GGPHNVVFVEDAI(*13C15N*)PK (+3)	529.29	550.33	y5	15.1	4			
			679.37	y6	13	2			
			778.44	y7	13.6	3			
			925.51	y8	14.7	1			
			1024.58	y9	16.9	5			
Phaeo Plastocyanin 1	GDSITWINNK(+2)	574.29	488.28	y4	22.6	3	17	0.1	1.5
			674.36	y5	20.3	2			
			775.41	y6	18.9	1			
			888.49	y7	19.6	4			
			975.53	y8	16.6	5			
	GDSITWI(*13C15N*)NNK (+2)	577.80	495.30	y4	22.6	3			
			681.38	y5	20.3	2			
			782.43	y6	18.9	1			
			895.51	y7	19.6	4			
			982.54	y8	16.6	5			
Phaeo Flavodoxin	AWIAQIK(+2)	415.25	258.12	b2	10.3	2	17.9	0.3	1.1
			388.26	y3	15.7	4			
			459.29	y4	15.1	3			
			572.38	y5	14	1			
			758.46	y6	16.9	5			
	AWIAQI(*13C15N*)K (+2)	418.76	258.12	b2	10.3	2			
			395.27	y3	15.7	4			
			466.31	y4	15.1	3			
			579.39	y5	14	1			
			765.47	y6	16.9	5			

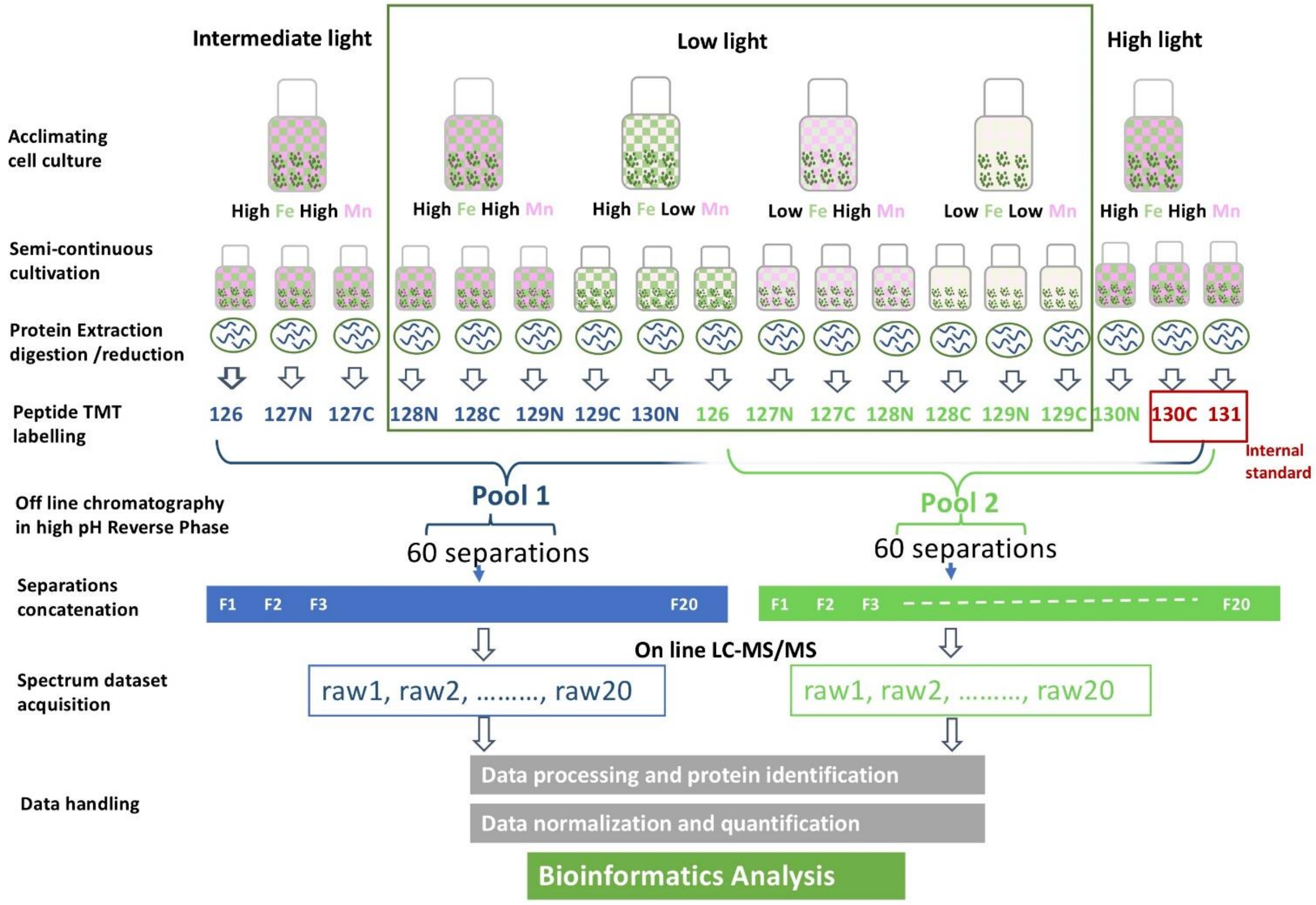

Supplementary Figure 1: Schematic overview showing culture experiment design and proteomic approach

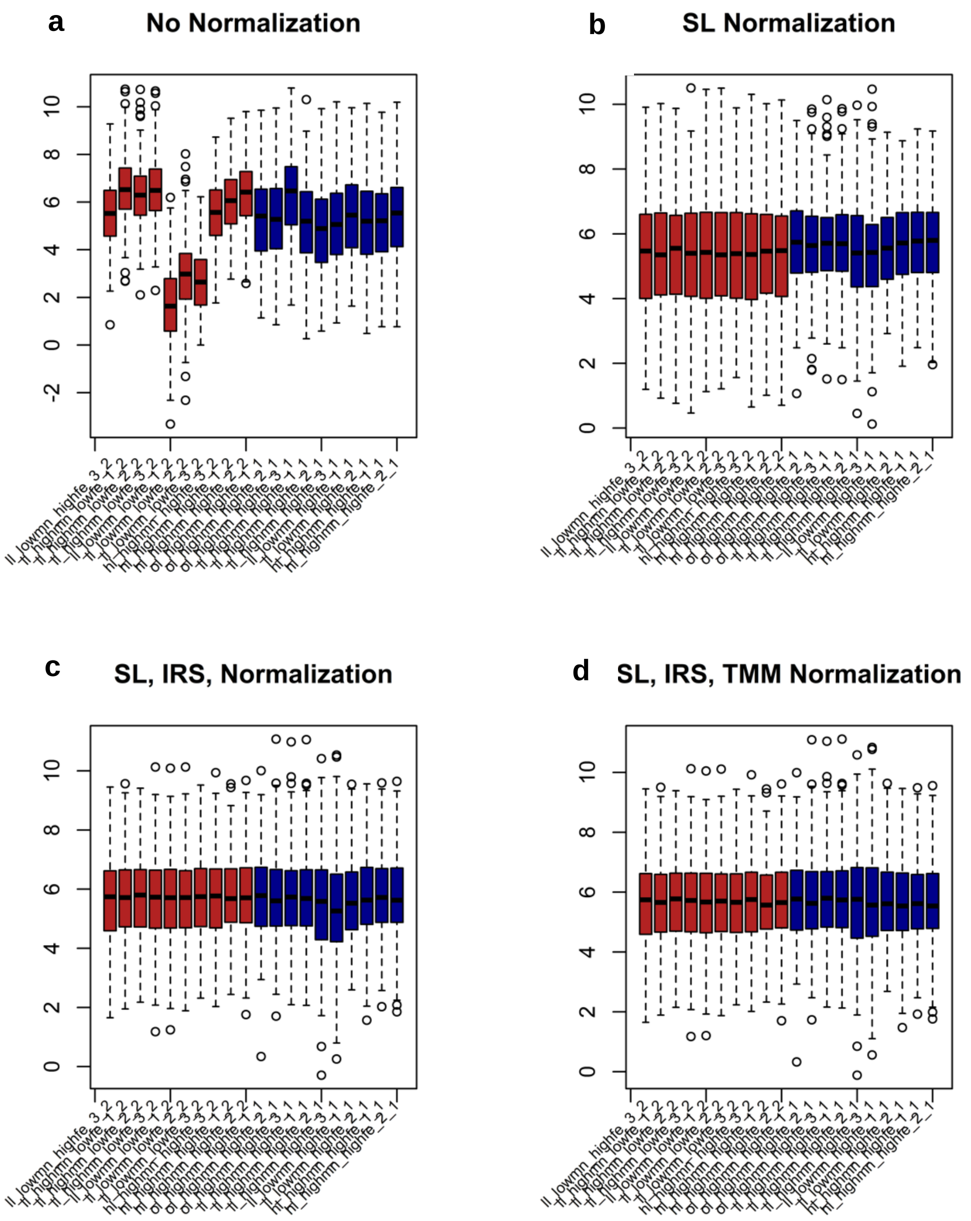

Supplementary Figure 2: Protein intensity distributions displayed as boxplots for each treatment, for each normalization step. Panel A corresponds to no normalization (raw intensity values), panel B shows the intensity distributions after sample loading normalization, panel C is after internal reference scaling (where the internal reference channels are 'hl_highmn_highfe_[1,2]_[1,2]. X-axis labels have the following information: light environment (low light or high light), Mn condition, Fe condition, biological replicate, MS-run. Colours correspond to different MS-runs. Source data are provided in Supplementary Data 1 and code for reproducing these analyses can be found at https://github.com/bertrand-lab/phaeo-mn-fe

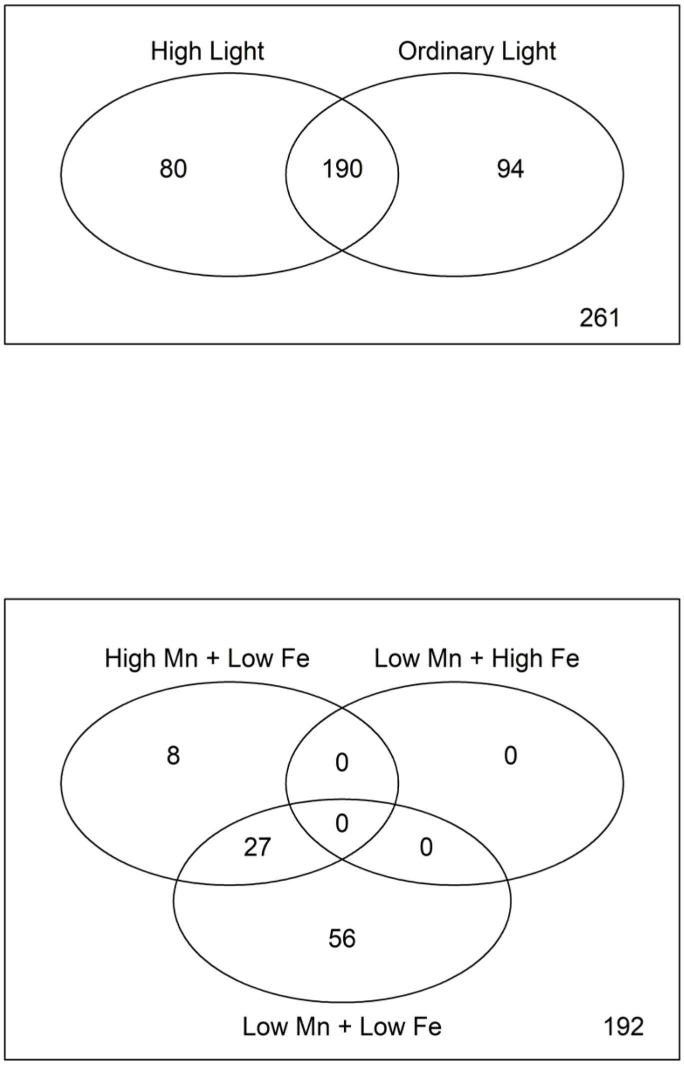

Supplementary Figure 3: Top: Venn diagram of differentially expressed genes comparing metal replete treatments. Differential expression in this diagram is compared to the low light condition. Bottom: Venn diagram of differentially expressed genes comparing low light treatments. Differential expression in this diagram is compared to the replete Fe/Mn condition. Significant differences were determined using empirical Bayes quasi-likelihood F-test (with edgeR), where n = 3 biologically independent samples per treatment. Source data are provided in Supplementary Data 1.

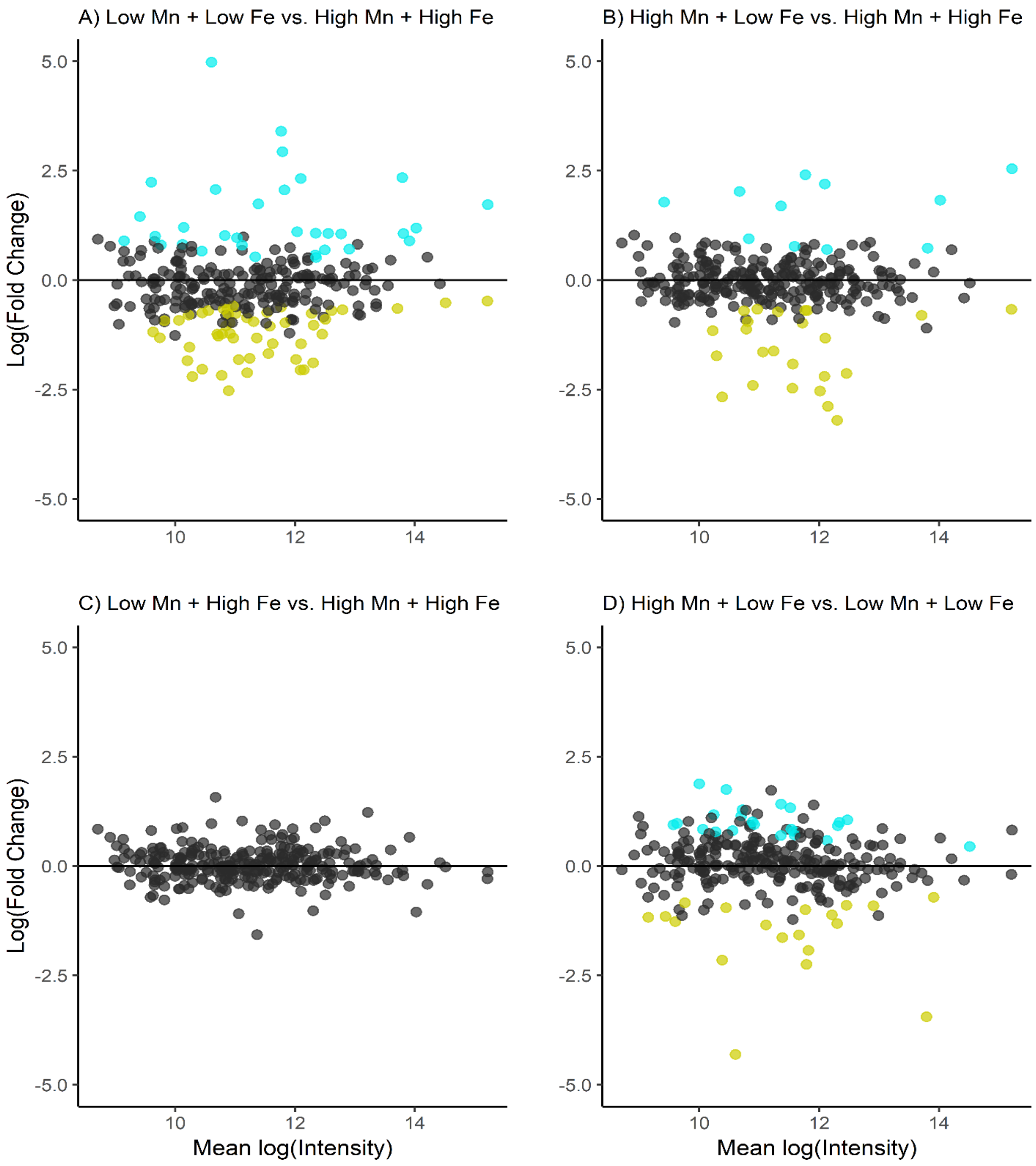

Supplementary Figure 4: Scatterplots of the mean natural logarithm of peptide intensity versus the natural logarithm of fold change. Panels A-C compare treatments with Mn/Fe replete as the baseline, and panel D highlights one treatment comparing to Mn/Fe deplete. Differentially expressed genes are coloured, with cyan being significantly upregulated compared to baseline and yellow being significantly downregulated compared to baseline. Significant differences were determined using empirical Bayes quasi-likelihood F-test (with edgeR), where n = 3 biologically independent samples per treatment. Source data are provided in Supplementary Data 1.

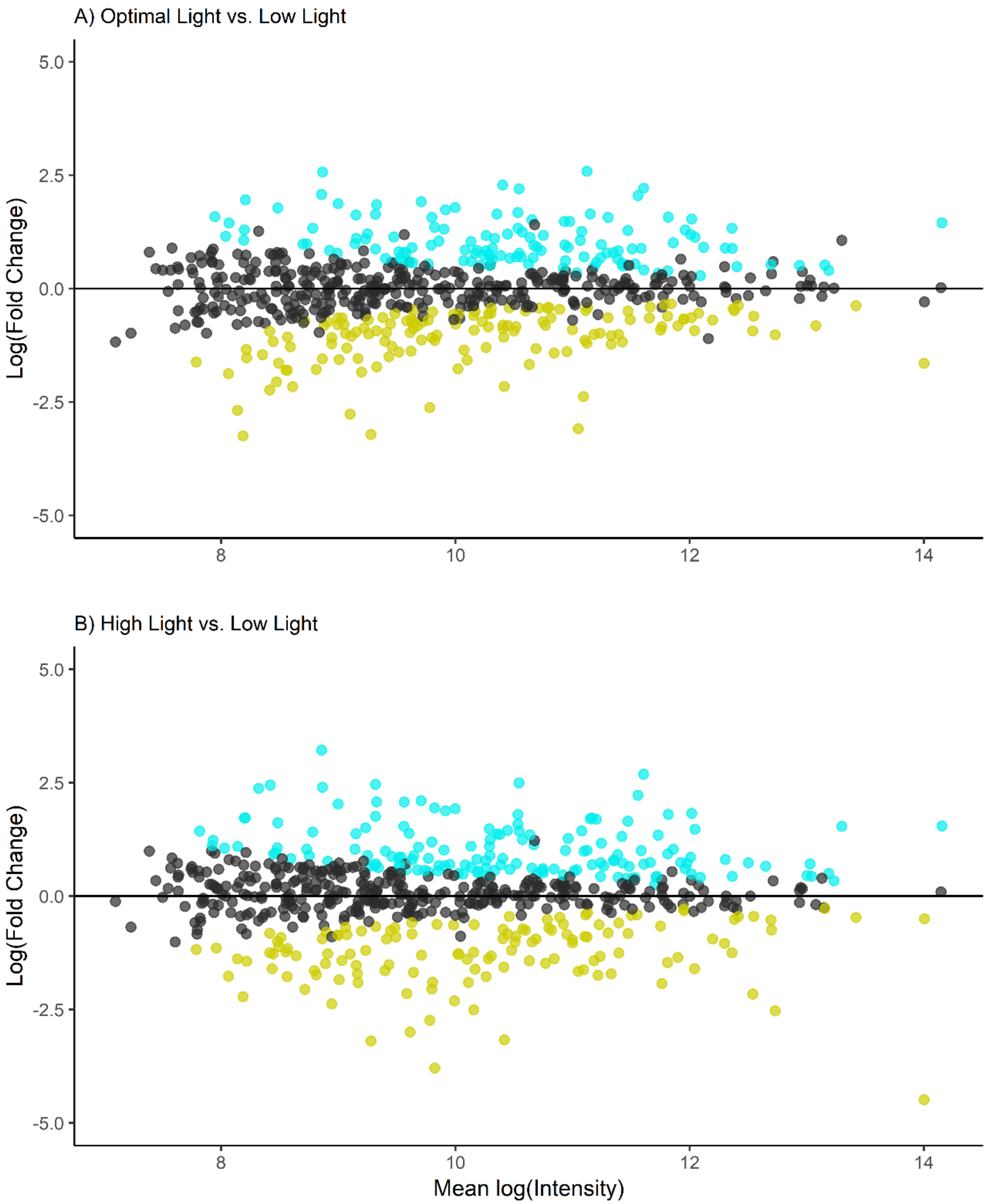

Supplementary Figure 5: Scatterplots of the mean natural logarithm of peptide intensity versus the natural logarithm of fold change. Panel A compares optimal light to low light, and panel B compares high light to low light. Differentially expressed genes are colored, with cyan being significantly upregulated compared to baseline and yellow being significantly downregulated compared to baseline. Significant differences were determined using empirical Bayes quasi-likelihood F-test (with edgeR), where n = 3 biologically independent samples per treatment. Source data are provided in Supplementary Data 1.

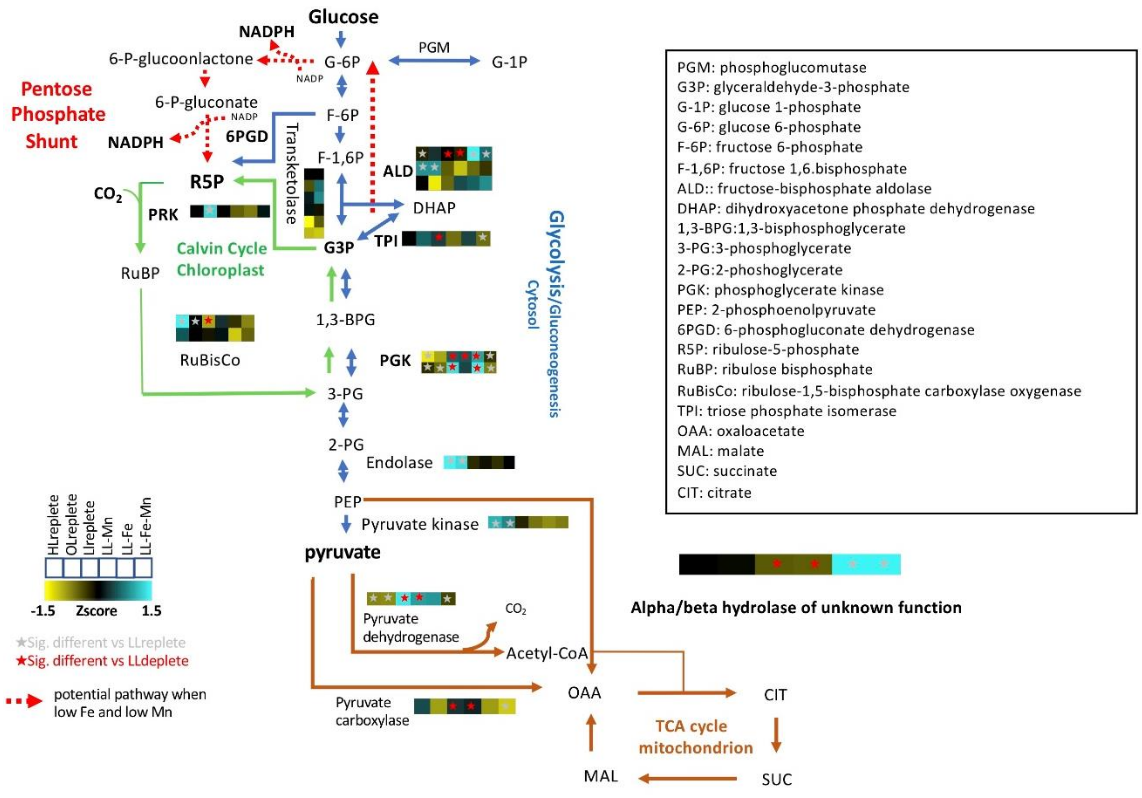

Supplementary Figure 6: Carbon metabolism changes induced by differences in light and trace metal availability. Mean standard scores for each treatment are shown and significant differences between treatments are indicated with red or gray stars. Proteins with multiple expression patterns had one or more subunits or isoforms detected. Significant differences were determined using empirical Bayes quasi-likelihood F-test where n = 3 biologically independent samples per treatment. Source data are provided in Supplementary Data 1.

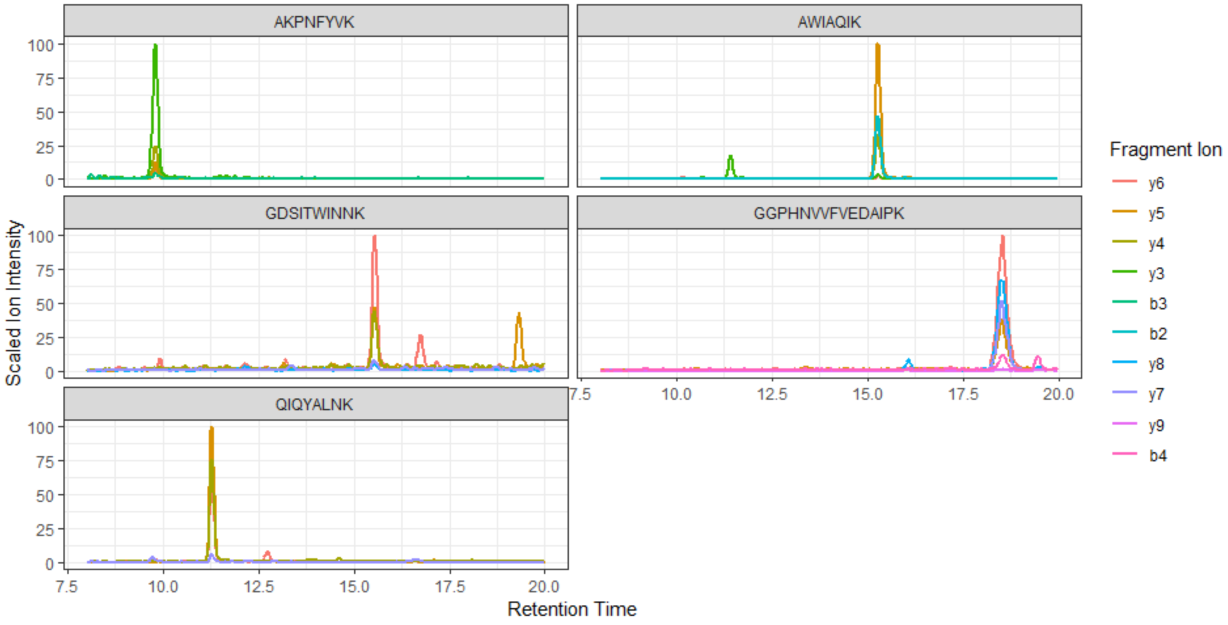

Supplementary Figure 7: Product ion chromatograms for the five key *Phaeocystis* peptides in the McMurdo Sound sample, Dec 28 2014. Ions and mass spectrometry parameters are described in Table S2 and peptides are described in Table 1.

Supplementary References

1. Perkins, A. Nelson, K. J., Parsonage, D., Poole, P. A. K. Peroxiredoxins: guardians against oxidative stress and modulators of peroxide signaling. *Trends Biochem. Sci.* **40,** 435–445 (2015).
2. Serrato, A. J., Fernández-Trijueque, J., Barajas-López, J.-D., Chueca, A. & Sahrawy, M. Plastid thioredoxins: a "one-for-all" redox-signaling system in plants. *Front. Plant Sci.* **4,** 463 (2013).
3. Kikutani, S. *et al.* Redox regulation of carbonic anhydrases via thioredoxin in chloroplast of the marine diatom Phaeodactylum tricornutum. *J. Biol. Chem.* **287,** 20689–700 (2012).
4. Richter, A. S., Pérez-Ruiz, J. M., Cejudo, F. J. & Grimm, B. Redox-control of chlorophyll biosynthesis mainly depends on thioredoxins. *FEBS Lett.* (2018). doi:10.1002/1873-3468.13216
5. Miziorko, H. M. Phosphoribulokinase: current perspectives on the structure/function basis for regulation and catalysis. *Adv. Enzymol. Relat. Areas Mol. Biol.* **74,** 95–127 (2000).
6. Mills, M. M. *et al.* Photophysiology in Two Southern Ocean Phytoplankton Taxa: Photosynthesis of Phaeocystis Antarctica (Prymnesiophyceae) and Fragilariopsis Cylindrus (Bacillariophyceae) under Simulated Mixed-Layer Irradiance1. *J. Phycol.* **46,** 1114–1127 (2010).
7. Bender, S. J. *et al.* Colony formation in Phaeocystis antarctica: connecting molecular mechanisms with iron biogeochemistry. *Biogeosciences* **15,** 4923–4942 (2018).
