# Supplementary material for: Manganese and iron deficiency in Southern Ocean Phaeocystis antarctica populations revealed through taxon-specific protein indicators
Source: Nat Commun. 2019 Aug 8;10:3582. doi: 10.1038/s41467-019-11426-z (PMC6687791; doi:10.1038/s41467-019-11426-z)
Supplement: Supplementary file 3 — Supplementary Files [file 41467_2019_11426_MOESM3_ESM.pdf]

Description of Additional Supplementary Files

File Name: Supplementary Data 1
Description: .xlsx sheet with the 280 quantified proteins with TMM scores, annotations, and differential expression analysis results as well as protein annotations and confidence scores for all identified proteins

File Name: Supplementary Data 2
Description: .xlsx sheet with all culture-based peptide and protein identifications, output from Proteome Discoverer.
