# Supplementary material for: Manganese and iron deficiency in Southern Ocean Phaeocystis antarctica populations revealed through taxon-specific protein indicators
Source: Nat Commun. 2019 Aug 8;10:3582. doi: 10.1038/s41467-019-11426-z (PMC6687791; doi:10.1038/s41467-019-11426-z)
Supplement: Supplementary file 6 — Reporting Summary [file 41467_2019_11426_MOESM6_ESM.pdf]

nature research

Corresponding author(s): Erin Bertrand

Last updated by author(s): Jun 26, 2019

Reporting Summary

Nature Research wishes to improve the reproducibility of the work that we publish. This form provides structure for consistency and transparency in reporting. For further information on Nature Research policies, see Authors & Referees and the Editorial Policy Checklist.

Statistics

For all statistical analyses, confirm that the following items are present in the figure legend, table legend, main text, or Methods section.

n/a	Confirmed	
☐	☒	The exact sample size (*n*) for each experimental group/condition, given as a discrete number and unit of measurement
☐	☒	A statement on whether measurements were taken from distinct samples or whether the same sample was measured repeatedly
☐	☒	The statistical test(s) used AND whether they are one- or two-sided<br>*Only common tests should be described solely by name; describe more complex techniques in the Methods section.*
☐	☒	A description of all covariates tested
☐	☐	A description of any assumptions or corrections, such as tests of normality and adjustment for multiple comparisons
☐	☒	A full description of the statistical parameters including central tendency (e.g. means) or other basic estimates (e.g. regression coefficient) AND variation (e.g. standard deviation) or associated estimates of uncertainty (e.g. confidence intervals)
☐	☒	For null hypothesis testing, the test statistic (e.g. *F*, *t*, *r*) with confidence intervals, effect sizes, degrees of freedom and *P* value noted<br>*Give P values as exact values whenever suitable.*
☐	☒	For Bayesian analysis, information on the choice of priors and Markov chain Monte Carlo settings
☐	☒	For hierarchical and complex designs, identification of the appropriate level for tests and full reporting of outcomes
☒	☐	Estimates of effect sizes (e.g. Cohen's *d*, Pearson's *r*), indicating how they were calculated

Our web collection on statistics for biologists contains articles on many of the points above.

Software and code

Policy information about availability of computer code

Data collection	Proteome Discoverer is publicly available and was used to generate and preliminarily process mass spectrometry data
Data analysis	Skyline was used to process targeted mass spectrometry data. Post-processing of mass spectrometry data was done with custom scripts provided on our github page and linked in the code accessibility statement. https://github.com/bertrand-lab/phaeo-mn-fe

For manuscripts utilizing custom algorithms or software that are central to the research but not yet described in published literature, software must be made available to editors/reviewers. We strongly encourage code deposition in a community repository (e.g. GitHub). See the Nature Research guidelines for submitting code & software for further information.

Data

Policy information about availability of data

All manuscripts must include a data availability statement. This statement should provide the following information, where applicable:

- Accession codes, unique identifiers, or web links for publicly available datasets
- A list of figures that have associated raw data
- A description of any restrictions on data availability

Mass spectrometry data are available via ProteomeXchange with accession number: PXD010974. Processed mass spectrometry data are available in Supplementary Data 1 and 2. Field mass spectrometry data will also be available through the Ocean Protein Portal at https://proteinportal.whoi.edu. The source data underlying Figures 1, 2B, 3, 4 and 5 are provided as a Source Data File. The source data underlying Figure 2A and Supplementary Figures 2-6 are provided in Supplementary Data 1.

Field-specific reporting

Please select the one below that is the best fit for your research. If you are not sure, read the appropriate sections before making your selection.

☐ Life sciences ☐ Behavioural & social sciences ☒ Ecological, evolutionary & environmental sciences

For a reference copy of the document with all sections, see nature.com/documents/nr-reporting-summary-flat.pdf

Ecological, evolutionary & environmental sciences study design

All studies must disclose on these points even when the disclosure is negative.

Study description	This study began with an algal culture-based assessment of Phaeocystis antarctica's responses to iron and manganese stress under low light availability. We used a factorial design to do this, +/- Fe and +/- Mn, with three independent replicates per treatment.We also cultured these algae with +Fe and +Mn under high and moderate light levels. The protein expression patterns induced by these treatments were assessed by mass spectrometry- based proteomics. These culture experiments were combined with field experiments that examined specific protein expression patterns and chlorophyll responses to the addition of trace metals (factorial design, addititon of metals to triplicate bottles +/- Fe and +/-Mn).
Research sample	Phaeocystis antarctica cultures- this organism is a dominant cultivable member of Southern Ocean phytoplankton communities. It is available from public culture repositories.<br>Field samples were collected in the coastal Ross Sea, McMurdo Sound and were chosen to represent the coastal Ross Sea
Sampling strategy	Triplicate samples for each experimental manipulation were performed; triplicates were chosen to balance the need for replication with the laborious nature of conducting trace metal clean semicontinuous culturing.
Data collection	Culture samples were collected and processed via filtration by M. Wu. Field samples were collected and processed by E. Bertrand, A. Allen and J. Hoffman in the field and E. Rowland and J. McCain. In depth collection procedures are described in the manuscript text
Timing and spatial scale	Field samples were collected over a one month period, approximately once a week, determined by practical constraints of weather and when it was safe to access the field site via helicopter.<br>Sampling of cultures was determined by culture behavior- cells were harvested once they came into steady-state (growth rate changing by less than 10% for three consecutive sampling events)
Data exclusions	No data were excluded from these analyses
Reproducibility	Culturing results were reproduced in small- scale cultures conducted to identify proper conditions for the metal treatments. The proteomics approach was conducted in triplicate but not independently replicated.
Randomization	mass spectrometry injections were randomly ordered.
Blinding	Most physiological measurements were conducted in a blinded fashion, with the analyst not having access to sample IDs during measurements for cell counts, chlorophyll etc. Proteomic data analysis was conducted with the analyst not having knowledge of the sample IDs in most cases.

Did the study involve field work? ☒ Yes ☐ No

Field work, collection and transport

Field conditions	Samples were collected from the sea ice edge in fair weather; air temperatures ranged from -10C to +10C and water temperature at depth of collection remained between -0.9 to -1.2 C
Location	Sea surface microbial community samples were acquired at the sea ice edge in McMurdo Sound of the Ross Sea from the same location (-77.62 S, 165.41 E) between Dec 28 2014 and Jan<br>22, 2015. Large volume protein samples (150-200L) were acquired from 1 m depth on Dec 28, Jan<br>6, Jan 15 and Jan 22
Access and import/export	Samples were acquired with permitting and processes required by the US Antarctic Program
Disturbance	Following US Antarctic program requirements to avoid marine macrofaunal disturbance at the sea ice edge

Reporting for specific materials, systems and methods

We require information from authors about some types of materials, experimental systems and methods used in many studies. Here, indicate whether each material, system or method listed is relevant to your study. If you are not sure if a list item applies to your research, read the appropriate section before selecting a response.

Materials & experimental systems

n/a	Involved in the study
☒	☐ Antibodies
☒	☐ Eukaryotic cell lines
☒	☐ Palaeontology
☒	☐ Animals and other organisms
☒	☐ Human research participants
☒	☐ Clinical data

Methods

n/a	Involved in the study
☒	☐ ChIP-seq
☐	☒ Flow cytometry
☒	☐ MRI-based neuroimaging

Flow Cytometry

Plots

Confirm that:

☐ The axis labels state the marker and fluorochrome used (e.g. CD4-FITC).

☐ The axis scales are clearly visible. Include numbers along axes only for bottom left plot of group (a 'group' is an analysis of identical markers).

☐ All plots are contour plots with outliers or pseudocolor plots.

☐ A numerical value for number of cells or percentage (with statistics) is provided.

Methodology

Sample preparation	acid dissolution of colonies then live introduction to flow cytometer
Instrument	BD Accuri C6
Software	BD Accuri software v 1.0
Cell population abundance	Purity of cultures was confirmed via fluorescence microscopy paired with DAPI staining
Gating strategy	Forward scattered light and chlorophyll a autofluorescence were used for gating, gating was done manually using a culture verified for purity as described above. Gating included >90% of all events from all cultures.

☐ Tick this box to confirm that a figure exemplifying the gating strategy is provided in the Supplementary Information.
